# CNV Hotspots in Testicular Seminoma Tissue and Seminal Plasma

**DOI:** 10.3390/cancers14010189

**Published:** 2021-12-31

**Authors:** Dora Raos, Irena Abramović, Miroslav Tomić, Alen Vrtarić, Tomislav Kuliš, Marijana Ćorić, Monika Ulamec, Ana Katušić Bojanac, Davor Ježek, Nino Sinčić

**Affiliations:** 1 Department of Medical Biology, School of Medicine, University of Zagreb, 10000 Zagreb, Croatia; dora.raos@mef.hr (D.R.); irena.abramovic@mef.hr (I.A.); mcoric1@kbc-zagreb.hr (M.Ć.); ana.katusic@mef.hr (A.K.B.); 2 Scientific Group for Research on Epigenetic Biomarkers, School of Medicine, University of Zagreb, 10000 Zagreb, Croatia; tkulis@kbc-zagreb.hr (T.K.); monika.ulamec@kbcsm.hr (M.U.); 3 Scientific Centre of Excellence for Reproductive and Regenerative Medicine, School of Medicine, University of Zagreb, 10000 Zagreb, Croatia; davor.jezek@mef.hr; 4 Department of Urology, University Clinical Hospital Centre “Sestre Milosrdnice”, 10000 Zagreb, Croatia; miroslav.tomic@kbcsm.hr; 5 Department of Clinical Chemistry, University Clinical Hospital Centre “Sestre Milosrdnice”, 10000 Zagreb, Croatia; alen.vrtaric@kbcsm.hr; 6 Department of Urology, University Hospital Centre Zagreb, 10000 Zagreb, Croatia; 7 Department of Pathology and Cytology, University Hospital Centre Zagreb, 10000 Zagreb, Croatia; 8 Ljudevit Jurak Clinical Department of Pathology and Cytology, University Clinical Hospital Centre “Sestre Milosrdnice”, 10000 Zagreb, Croatia; 9 Department of Pathology, University of Zagreb School of Medicine, 10000 Zagreb, Croatia; 10 Department of Histology and Embryology, University of Zagreb School of Medicine, 10000 Zagreb, Croatia

**Keywords:** testicular seminoma, liquid biopsy, CNV, biomarkers

## Abstract

**Simple Summary:**

Testicular seminoma represents the most common type of testicular germ cell tumours, which are the most prevalent malignancies among the male population in reproductive age. Thus, it is crucial to find novel biomarkers for early detection and improve patient management. Copy number variation (CNV) is associated with various cancers including seminoma. Therefore, the current study aims to investigate CNV of specific genes and determine their potential as a possible seminoma biomarker. CNVs were investigated in genomic DNA from seminoma tissue, as well as in cell-free DNA (cfDNA) from seminal plasma as liquid biopsy. We detected increased CNVs in tissue samples, as well as in cfDNA from seminal plasma. According to obtained data, seminoma CNV hotspots are present and are reflected in seminal plasma. Although clinical value is yet to be determined, presented data emphasize a potential use of CNV as an SE biomarker.

**Abstract:**

Seminoma (SE) is the most frequent type of testicular tumour, affecting predominantly young men. Early detection and diagnosis of SE could significantly improve life quality and reproductive health after diagnosis and treatment. Copy number variation (CNV) has already been associated with various cancers as well as SE. In this study, we selected four genes (*MAGEC2*, *NANOG*, *RASSF1A*, and *KITLG*) for CNV analysis in genomic DNA (gDNA), which are located on chromosomes susceptible to gains, and whose aberrant expression was already detected in SE. Furthermore, CNV was analysed in cell-free DNA (cfDNA) from seminal plasma. Analysis was performed by droplet digital polymerase chain reaction (ddPCR) on gDNA from SE and nonmalignant testicular tissue. Seminal plasma cfDNA from SE patients before and after surgery was analysed, as well as from healthy volunteers. The CNV hotspot in gDNA from SE tissue was detected for the first time in all analysed genes, and for two genes, *NANOG* and *KITLG* it was reflected in cfDNA from seminal plasma. Although clinical value is yet to be determined, presented data emphasize a potential use of CNV as a potential SE biomarker from a liquid biopsy.

## 1. Introduction

Testicular germ cell tumours (TGCT) are the most common malignancies in young Caucasian men between the ages of 15 to 40 years [1]. Even though mortality is decreasing in most countries, the incidence of TGCT has increased over the last decades [2], whereas the European ancestry is five times more likely to develop TGCT than African and Asian, thus indicating a genetic predisposition for this disease [3]. All TGCT arises from the same precursor lesion, the germ cell neoplasia in situ (GCNIS) [4], but based on their degree of differentiation and histological characteristics, TGCT are divided into seminomas (SE) and nonseminomas. SE represents approximately 55% of all TGCT cases [5].

Although environmental exposure has a great impact on TGCT development [6], the genetic contribution to the pathogenesis of TGCT is well known. Familial genetics is highly associated with TGCT development [7]. Indeed, brothers of TGCT patients have 8 to 10 times, fathers 4 times, and sons 6 times higher risk of developing the disease compared to the general population [8]. Additional genetic material on the 12p chromosome is common in TGCT but not detected in GCNIS [9]. This means that this amplification of 12p plays a critical role in TGCT progression. Genome-wide association studies have discovered single nucleotide polymorphism (SNP) of several genes, such as *KITLG*, *SPRY4*, *BAK1*, *DMRT1*, *TERT*, and *ATF7IP* to be associated with TGCT risk [10,11,12]. The strongest association was found for *KITLG* where the risk for TGCT was increased 2.5-fold.

In addition to SNP, gene copy number variations (CNVs) have been associated with the susceptibility of various cancers as well. CNV is the gain or loss of DNA which can range from small microscopic events to the aneuploidy of the whole chromosome [13]. Multiple genes influence cancerogenesis, so the interaction among CNVs of different genes associated with cancer susceptibility modulates (decreases or increases) the risk of sporadic cancer in individuals [14]. This means that detecting CNVs specific for a particular type of cancer could enable a risk estimation for each individual and the use of CNV as a patient management biomarker. Still, there are only a few publications that present data on CNVs association with TGCT.

Based on the literature review, we selected genes located on chromosomes (Table 1) susceptible to gains and investigated the presence of CNVs. Indeed, the expression of selected genes, possibly related to existing CNVs, was reported altered in SE. *NANOG* is an embryonal marker already used as an additional biomarker on the protein level in SE diagnosis [15]. *KITLG* takes part in the *KIT-KITLG* signal pathway, which is a central pathway in TGCT tumorigenesis [16]. *MAGEC2* is a cancer-testis gene, for which disturbed protein expression is associated with SE [5]. *RASSF1A* is a tumour-suppressor gene whose methylation is altered in various cancers [17]. However, it is located on chr. 3, on which gains were detected in testicular primary seminoma [18].

In the diagnostics of TGCT, serum biomarkers like alpha-fetoprotein, beta-human chorionic gonadotropin, and lactate dehydrogenase represent a valuable tool. However, these biomarkers detect nonseminoma better than SE. In the case of SE, serum biomarkers are either slightly elevated or not elevated at all [19], which further complicates the diagnostic process of SE. Therefore, investigation of CNVs was performed on SE only, with the goal to identify possible benefits for diagnostic application based on a liquid biopsy concept. Cell-free DNA (cfDNA) is widely researched in the context of tumour biomarkers from liquid biopsy because it represents a non-invasive approach [20]. Seminal plasma could be a great source of potential biomarkers for TGCT due to anatomical reasons [21].

In this study, CNVs of *NANOG*, *KITLG*, *MAGEC2*, and *RASSF1A* were investigated in gDNA from SE and in cfDNA from seminal plasma of SE patients before and after surgery. The aim of this study was to analyse CNV, but further clarify whether detected CNVs in gDNA from SE are reflected in cfDNA from seminal plasma, as well as determine if CNV changes in cfDNA could be detected after seminoma surgery. If so, this would enable further development of SE diagnostics toward the liquid biopsy concept by using CNV as a diagnostic and prognostic SE biomarker.

## 2. Materials and Methods

### 2.1. Study Population

In this prospective study, twenty-four SE patients were recruited by University Hospital Centre “Sestre milosrdnice” (UHCSM) and the University Hospital Centre Zagreb (UHCZ). One set of ejaculates was collected before (preOP) and after surgery (postOP). Clinical pathologists based on histopathological analysis of obtained testicular tissue confirmed diagnosis of SE. As a control group, thirty-five healthy volunteers (HV) were included, and their ejaculate samples were collected. All participants were informed about the study and written consent was obtained from every participant before admission into the study. In addition, testicular tissue samples of twelve patients with non-malignant diagnoses (NTT) were retrieved from the UHCSM paraffin tissue archive as a control group for the gDNA analysis, diagnosed as testicular trauma or inflammatory disease. The study was conducted following the Declaration of Helsinki (2011) and performed under the approval of the Ethical Committees of UHCSM, UHCZ, and the University of Zagreb School of Medicine.

### 2.2. Sample Collection

#### 2.2.1. Ejaculate

Ejaculate samples were collected by masturbation after 3–5 days of sexual abstinence. After 30–60 min liquefaction at room temperature, samples were processed by dual centrifugation (at 400× *g* and 12,000× *g*, both for 10 min at room temperature) into seminal plasma. All samples were stored at −80 °C before further analysis.

#### 2.2.2. Seminoma Tissue

SE tissue samples from patients were collected by therapeutic radical orchiectomy. Samples were fixed, paraffin-embedded, and used for histopathologic diagnosis and staging. SE tissue samples and archive NTT samples were subjected to histopathological review. In the SE surrounding tissue of all samples, GCNIS was found. The proportion of GCNIS varied among the samples from 10–50% of SE surrounding parenchyma across sections. Areas containing only SE were determined for subsequent gDNA isolation. 

### 2.3. DNA Isolation and Quantification

GDNA from SE tissue was isolated according to the optimized protocol [22]. GDNA concentration was measured by a NanoDrop 2000c spectrophotometer.

CfDNA was isolated from seminal plasma, using the NucleoSnap cfDNA kit for cell-free DNA from plasma (MACHERY-NAGEL, Düren, Germany) and a vacuum pump from QUIAGEN (Hilden, Germany) to maximise cfDNA quantity and quality [23]. Volumes of ejaculate varied from 1.5 mL to 4 mL. Eluate volume after isolation was 100 μL. Quantification of cfDNA was performed using a Quant-iT™ PicoGreen^®^ dsDNA detection kit (Molecular Probes, Eugene, OR, USA) in triplicate. In addition, 3 μL of cfDNA final elution was mixed with PicoGreen reagent (THERMOFISHER SCIENTIFIC, Walthamu, MA, USA) according to the manufacturer’s instructions. The intensity of fluorescence was measured on a spectrofluorometer (TECAN, Männedorf, Switzerland).

Isolated gDNA from TCam-2 cell line (obtained from professor L. Looijenga, Erasmus University Medical Center, Rotterdam, Netherlands) was used as a reference sample for SE labelled as RCLS.

### 2.4. Digital Droplet PCR (ddPCR)

CNV in every sample was analysed using digital droplet PCR (ddPCR). Primers for target genes were commercial or designed in-house (Table 2). As a reference gene, *AP3B1* was used. Target primers were labelled with the fluorescent dye reporter FAM and reference primers with HEX. Briefly, the master mix for ddPCR included ddPCR Supermix for probes or EvaGreen Supermix (Bio-Rad, Hercules, CA, USA), restriction enzymes (Bio-Rad, Hercules, CA, USA), target primers, reference primers, RNase-free H2O, and a DNA sample. Twenty (20) µL of each ddPCR mixture and seventy (70) µL of Droplet Generation Oil for Probes or ddPCR Droplet Generation Oil for EvaGreen (Bio-Rad, Hercules, CA, USA) were loaded into the disposable DG8 Cartridge (Bio-Rad, Hercules, CA, USA) located in a cartridge holder (BioRad, Hercules, CA, USA). The cartridge was placed inside the QX200 Droplet Generator, covered with the DG8 Gasket (Bio-Rad, Hercules, CA, USA). In the QX200 droplet Generator, every sample was partitioned into 20,000 nanolitre-sized droplets, with the target sequences being randomly distributed into the droplets. After droplet generation, droplets were carefully transferred to a ddPCR plate (BioRad, Hercules, CA, USA) and heat-sealed in the PX1 PCR Plate Sealer (Bio-Rad, Hercules, CA, USA) with a pierceable aluminum foil (Bio-Rad, Hercules, CA, USA). PCR amplification was executed on the CFX96 Deep Well PCR thermal cycler (BioRad, Hercules, CA, USA) under the following thermal conditions (Table 2). In every assay, no template control (NTC) was included. The cycled plate was transferred and read in the FAM and HEX channels using the QX200 reader (Bio-Rad, Hercules, CA, USA). The manual threshold for target genes and *AP3B1* were applied based on NTC. Copy number values for target genes were subsequently obtained from the ratio of target molecule concentration and *AP3B1* molecule concentration multiplied by the number of *AP3B1* copies (2) in the genome. The distribution of the obtained CNV is shown in Table S1.

### 2.5. Statistical Analysis

Obtained values are divided into two groups. In the first group, the number of gene copies are raw values with two decimal places and, in the second group, the number of gene copies is value rounded to integers. Non-parametric tests were performed to compare CNV between analysed groups. Mann-Whitney was used for comparison between two groups where samples were not paired, while the Wilcoxon matched-pairs signed rank test was used to compare groups consisting of paired samples. For comparison, rounded values of CNV between RCLS and SE tissue Wilcoxon signed rank test were used. All obtained data are shown with a scatter dot-plot. A *p*-value < 0.05 (two-tailed) was considered statistically significant.

## 3. Results

Based on Tumour, Node, and Metastasis (TNM) classification [24], from a total of 24 enrolled SE patients, 17 had T1, 5 T2, and 1 T3. For one patient, TNM data were missing. An age difference between healthy volunteers and SE patients was detected (*p* < 0.001). The clinical characteristics of the study groups are summarized in Table 3.

### 3.1. CNV in Tissue Samples

**Figure 1:**
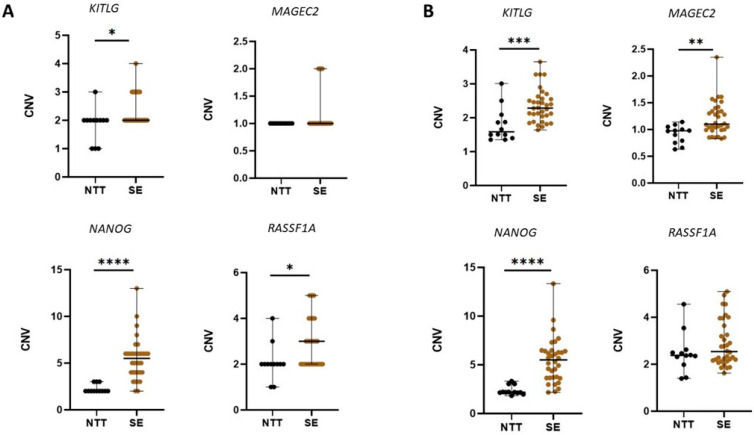
Copy number variation (CNV) detected in non-malignant and seminoma tissue. (**A**) For each analysed gene, the rounded number of CNV in gDNA is presented. (**B**) For each analysed gene, raw data of CNV detected in gDNA are presented. Black lines represent median with interquartile range. A statistically significant difference is indicated as * *p* < 0.05, ** *p* < 0.01, *** *p* < 0.001, and **** *p* < 0.0001. NTT, non-malignant diagnoses; SE, seminoma.

The comparison of detected CNVs in NTT and SE tissue is shown in Figure 1. Raw data, as well as round numbers of CNV, are shown. The levels of CNV in selected genes were not normally distributed in subjects within the same group. Hence, they are presented by quartile range. The statistically significant difference in CNV between NTT and SE was detected in all analysed genes.

A separate analysis of genes revealed that CNV of *NANOG* was the most statistically significant difference between NTT and SE. In SE, detected CNV gain of *NANOG* varied from CNV = 3 (11.76%) to CNV = 13 (2.93%). In most SE samples, CNV = 6 (32.35%), CNV = 5 (14.71%), or CNV = 4 (14.71%). CNV = 8; 9; 10 was detected in just 2.94% of SE. In case of *KITLG*, CNV gains were detected, in 26.47%, SE samples detected CNV = 3, while 2.94% detected CNV = 4. Regarding *RASSF1A*, gains of this gene were also detected, i.e., we observed CNV= 3 in 26.27%, CNV= 4 in 14.71%, and CNV= 5 in 11.76% SE samples. For *MAGEC2*, increased CNV, i.e., CNV = 2 was detected in only 17.65% of SE. All performed statistical tests with corresponding *p*-values are shown in Table S2.

### 3.2. CNV in cfDNA from Seminal Plasma

**Figure 2:**
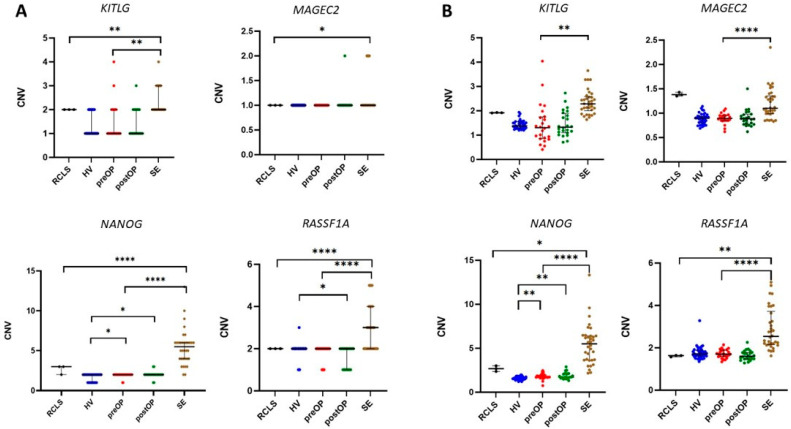
Copy number variation detected in gDNA from the referent cell line for seminoma, cfDNA from seminal plasma, and gDNA from seminoma tissue. (**A**) For each analysed gene, the rounded number of CNV detected in gDNA and cfDNA is presented. (**B**) For each analysed gene, raw data of CNV detected in gDNA and cfDNA are presented. Black lines represent median with interquartile range. A statistically significant difference is indicated as * *p* < 0.05, ** *p* < 0.001, and **** *p* < 0.0001. SE, seminoma; HV, healthy volunteer.

Comparing data obtained on cfDNA from preoperative seminal plasma and gDNA from SE tissue, a statistically significant difference was detected of CNVs of all analysed genes, except *MAGEC2* (Figure 2). Copy number gain was detected for *KITLG*, i.e., in 4.16% of preoperative samples CNV = 3 or CNV = 4 and for *NANOG* (CNV = 3 in 8.33%). However, in the case of *KITLG*, copy number loss was also detected, i.e., in 62.5% of preoperative samples, CNV = 1. The copy number loss was also observed in preoperative samples for *RASSF1A* (CNV = 1 in 16.6%) and *NANOG* (CNV = 1 in 8.33%) but in fewer samples than it was for *KITLG*.

In cfDNA from postoperative seminal plasma samples, aberrant CNV was detected for all analysed genes, i.e., *MAGEC2* (CNV = 2 in 4.16%), *RASSF1A* (CNV = 1 in 37.5%), *NANOG* (CNV = 3 in 8.33%; CNV = 1 in 8.33%), and in *KITLG* (CNV = 1 in 62.5%; CNV = 3 in 4.16%) in comparison to SE gDNA. However, these results were not statistically significant.

Comparing CNV in cfDNA from HV, preoperative, and postoperative seminal plasma samples, the following results were obtained. CNV of *NANOG* significantly differed between HV and preoperative samples, and CNV gain of *NANOG* was detected in 8.33% (CNV = 3). For other genes, differences between HV and preoperative samples were not detected. In the case of HV vs. postoperative, a significant CNV decrease in postoperative samples was detected for *RASSF1A*, and an increase was detected for *NANOG*. In comparison between preoperative and postoperative seminal plasma samples, no statistically significant difference in CNVs was detected for any analysed gene. In analysing data on preoperative and postoperative seminal plasma samples, no statistically significant difference in CNVs was detected for any analysed gene. However, although statistically not significant, a decrease in CNV trend in all selected genes was observed in postoperative samples compared to preoperative (Figure 2, Table S2).

In a comparison of CNV detected in RCLS and SE tissue, a statistically significant increase of CNV in all analysed genes was detected in SE tissue. The highest difference was observed in the case of *NANOG* and *RASSF1A* (Figure 2). All performed statistical tests with corresponding *p*-values are shown in Table S2.

## 4. Discussion

CNVs are associated with various tumours [25]. Defining CNVs associated with TGCT is of great importance because it could lead to earlier diagnosis and better patient management, thus promoting significant improvement in life quality and reproductive health of young men after diagnosis and treatment.

In the previous studies, gains and losses of specific chromosomal regions in TGCT were investigated [18,26,27,28,29,30] rather than CNVs of specific genes [31,32,33,34,35]. Therefore, we investigated CNVs of specific genes, located on the chromosomal regions with detected gains in SE. In addition, aberrant expression of selected genes in SE was reported [5]. The investigation was conducted not only on tissue samples but on seminal plasma samples as well. Detection of specific CNVs in seminal plasma could represent a non-invasive tool for early screening and management of SE patients. The advantage of seminal plasma as liquid biopsy is its direct contact with testicular tissue and primary tumour [36]. However, except from SE, cfDNA from other tissues like epididymis, seminal vesicles, prostate, etc. is released into the seminal fluid as well. Healthy tissue “contamination” indeed represents a potential limitation of this study. This challenge is addressed by study design where data obtained by analysis of seminal plasma from SE patients were compared to data from seminal plasma of healthy volunteers. By this design, we are certain that detected CNV alterations originate from SE and not from other male reproductive system tissues.

Strujik et al. investigated CNVs in SE and reported that no CNV hotspot in SE was detected [37]. However, we detected CNV gain of all analysed genes in SE tissue. This was expected because triploidy is a characteristic of SE, and obtained results are in line with that chromosomal anomaly [38]. The highest CNV was detected in *NANOG* which is in accordance with previous research [39] and its function in SE. Furthermore, a CNV of *KITLG* was also detected in SE tissue. These findings fit with prior observations and hypotheses regarding how each gene/pathway may modify TGCT risk. The *KIT* pathway has been suggested to be constitutively activated in human TGCTs as a result of gain-of-function mutations in the *KIT* oncogene and/or overexpression of *KIT* [40]. Shen et al. reported focal amplification of *KIT* in SE [34]. This CNV gain of *KIT*, as well as the CNV of *KITLG* reported here, may be related to the known activation of the *KIT* pathway in SE [41]. Next, *RASSF1A* is a tumour-suppressor gene, involved in the regulation of signalling pathways important for apoptosis, microtubule stability, and repression of the cell cycle [17]. *RASSF1A* was reported hypomethylated in SE and was concluded that its aberrant expression in SE is a consequence of aberrant DNA methylation [42]. However, in this study, increased CNV of *RASSF1A* was detected in SE tissue. It is clear that, in most SE samples, detected *RASSF1A* CNVs are reassembled around CNV = 2 and CNV = 3. In the case of *RASSF1A* CNV = 2 in SE tissue, it is logical to conclude that *RASSF1A* has no potential as SE biomarker because it is overlapping with CNVs detected in NTT. However, detected *RASSF1A* CNV gains in SE tissue, especially CNV = 3, could implicate that CNVs play a role in the already described altered expression of *RASSF1A* in SE. Congruently, increased CNV found in *MAGEC2* could be a basis for aberrant expression detected on the protein level as well [43].

Comparing cfDNA from seminal plasma and gDNA from SE tissue, a significant difference of CNV was detected. CNV of *NANOG*, *RASSF1A*, and *KITLG* was significantly lower in cfDNA from preoperative seminal plasma than in gDNA from SE tissue, as well as in cfDNA from postoperative. This could be because in seminal plasma cfDNA originates from SE, non-malignant testicular tissue, and sperm cells [44]. Sperm cells represent male germ cells with a haploid number of chromosomes [45]. Therefore, cfDNA from sperm cells with haploid chromosome number could camouflage the cfDNA that originates from the tumour with increased CNVs. In the analysis of cfDNA from seminal fluid, obtained results represent data on SE cfDNA, but a fraction of GCNIS cfDNA as well since GCNIS always accompanies SE. Genomic alterations are described to be present already in GCNIS, as later in SE [38]. Therefore, the presence of GCNIS cfDNA in semen does not preclude the conclusion that CNV in cfDNA of patients with SE may have biomarker potential.

Comparison between CNVs detected in cfDNA from seminal plasma of HV, preoperative, and postoperative samples disclosed that CNV of *NANOG* was increased in preoperative samples, which indicates the reflection of increased *NANOG* CNV from gDNA SE tissue in cfDNA from seminal plasma. The same was detected for *KITLG*. However, we also detected lower CNV values in a few preoperative compared to postoperative samples. Although detected in just a small number of samples, this fact represents unexpected data that could not be comprehensively explained based on previous research and available literature. It is reasonable to suspect that such a finding could be a reflection of heterogeneity of seminoma in preoperative samples but also the heterogeneity of healthy testis in postoperative samples as well. Furthermore, a decreased CNV trend detected for all selected genes in postoperative seminal plasma indicates that, with SE removal, CNV is normalizing and easily detectable in cfDNA from seminal plasma. This gives perspectives for future research of CNV potential as a biomarker for treatment success.

For all analysed genes, a significant difference in CNV was detected between referent DNA samples from TCam-2 cell line and gDNA from SE tissue. The possible explanation could be that TCam-2 cell culture originates from SE which did not have CNV. Indeed, our results show that not every SE contains CNV of analysed genes. Detected CNVs in SE can be explained by inheritance [46] or de novo CNV alterations [47]. As the SE patients are exposed to environmental factors, these could indeed contribute to the formation of de novo CNVs [48], with an impact on SE tumorigenesis.

Apart from valuable results presented in this study, certain study limitations should be highlighted. The presence of cfDNA in seminal fluid from other tissues than SE represents a limitation that cannot be surpassed since available technology does not enable preanalytical separation of cfDNA originating from tumours and other tissues, respectively. Furthermore, TGCT are a very heterogeneous group, and there is a need to investigate CNVs on nonseminoma as well. Comparison of SE and nonseminoma patients’ data could further test if CNV of selected genes indeed differs between TGCT components. In addition, the detected overlapping between control and SE patients’ data indicates that these results should be tested on the larger number of samples. Therefore, the clinical value of the presented data is limited, and further research is required.

## 5. Conclusions

In this study, we aimed to investigate whether CNVs of selected genes exist and are reflected in seminal fluid. For the first time, a CNV hotspot in SE tissue was detected for *KITLG*, *RAFSF1A*, and *MAGEC2*. In all four analysed genes, an increased CNV in SE tissue regarding NTT was detected. Furthermore, this is the first study on SE that disclosed information about CNV on cfDNA from seminal plasma and discovered that CNV gain of *NANOG* and *KITLG* from SE tissue is indeed reflected in cfDNA from seminal plasma. Although statistically not significant, a decrease in CNV trend in all four analysed genes in postoperative compared to preoperative seminal plasma samples indicates that operational treatment induces at least slight normalization in CNV to an HV level. Apart from the value of presented data in the attempt to detect new possible SE biomarkers from liquid biopsy, the lack of data from nonseminoma, as well as sample size requires further investigation to determine clinical value.

## Figures and Tables

**Table 1:** Chromosomal locations of selected genes.

Gene	Location
*RASSF1A*	3p21.31
*NANOG*	12p13.31
*KITLG*	12q21.32
*MAGEC2*	Xq27.2

**Table 2:** Primers used for CNV detection of selected genes.

**Gene**	**Primer**	**Sequence of the In-House Assay**	**Temperature (°C)**	**No. of Cycles**
*KITLG*	F	5′-GCGGGACTTGGGTCTCATTT-3′	57.5	40
	R	5′-TCTGGAGCCATGCAAATGGT-3′		
**Gene**	**Commercial Assay ID**		**Temperature (°C)**	**No. of Cycles**
*RASSF1A*	dHsaCNS143255910		57.5	40
*NANOG*	dHsaCNS193219338		57.5	40
*MAGEC2*	dHsaCNS241647353		57.5	40

**Table 3:** Clinicopathological data of patients included in the study.

Clinicopathological Variables		SE Patients (*n* = 24)	Healthy Volunteers (*n* = 35)
Median age, years (range)		35 (20–49)	26 (16–42)
TNM classification	T1	17	-
	T2	5	
	T3	1	
Median tumour size (range) cm		3.4 (0.3–8)	

## Data Availability

The data generated in this study are available within the article. Raw data for this study were generated at EpiMark, School of Medicine, University of Zagreb, UHCZ, and UHCSM. The raw data generated in this study are available upon request from the corresponding author except for patients’ personal data protected from the public by positive laws and regulations.

## Supplementary Materials

The following supporting information can be downloaded at: https://www.mdpi.com/article/10.3390/cancers14010189/s1. Table S1: Medians and range of obtained CNV values. Table S2: Statistical tests and obtained *p*-values.
