# Supplementary material for: CNV Hotspots in Testicular Seminoma Tissue and Seminal Plasma
Source: Cancers (Basel). 2021 Dec 31;14(1):189. doi: 10.3390/cancers14010189 (PMC8750740; doi:10.3390/cancers14010189)
Supplement: Supplementary file 1 [file cancers-14-00189-s001.zip › cancers-1464748-supplementary.pdf]

cancers

MDPI

Supplementary Materials

CNV Hotspots in Testicular Seminoma Tissue and Seminal Plasma

Dora Raos, Irena Abramović, Miroslav Tomić, Alen Vrtarić, Tomislav Kuliš, Marijana Ćorić, Monika Ulamec, Ana Katušić Bojanac, Davor Ježek and Nino Sinčić

Table S1. Medians and range of obtained CNV values.

Data	Groups	*MAGEC2*	*RASSF1A*	*NANOG*	*KITLG*
		**Median (Range)**	**Median (Range)**	**Median (Range)**	**Median (Range)**
Raw data of CNV	NTT	0.98 (0.63–1.14)	2.38 (1.39–4.56)	2.18 (1.83–3.32)	1.58 (1.64–3.01)
	SE tissue	1.1 (0.83–2.35)	2.53 (1.62–5.1)	5.5 (2.17–13.33)	2.29 (1.64–3.65)
	RCLS	1.38 (1.35–1.43)	1.63 (1.57–1.64)	2.68 (2.36–3.01)	1.92 (1.91–1.93)
	HV	0.89 (0.69–1.14)	1.71 (1.35–3.28)	1.56 (1.2–1.97)	1.38 (1.2–1.94)
	preOP	0.89 (0.62–1.09)	1.7 (1.34–2.14)	1.75 (1.32–2.89)	1.31 (0.41–4.04)
	postOP	0.89 (0.62–1.5)	1.6 (1.2–2.26)	1.73 (0.75–2.46)	1.33 (0.71–2.73)
Rounded number of CNV	NTT	1 (0)	2 (1–4)	2 (2–3)	2 (1–3)
	SE tissue	1 (1–2)	3 (2–5)	5.5 (2–13)	2 (2–4)
	RCLS	1 (0)	2 (0)	3 (2–3)	2 (0)
	HV	1 (0)	2 (1–3)	2 (1–2)	1 (1–2)
	preOP	1 (0)	2 (1–2)	2 (1–2)	1 (1–4)
	postOP	1 (1–2)	2 (1–2)	2 (1–3)	2 (2–4)

NTT, non-malignant diagnoses; SE, seminoma.; HV, healthy volunteer.

Table S2. Statistical tests and obtained *p*-values.

Performed Test	Groups	*MAGEC2*		*RASSF1A*		*NANOG*		*KITLG*	
		**Raw Data**	**Rounded Value**	**Raw Data**	**Rounded Value**	**Raw Data**	**Rounded Value**	**Raw Data**	**Rounded Value**
		***p* Value**	***p* Value**	***p* Value**	***p* Value**	***p* Value**	***p* Value**	***p* Value**	***p* Value**
Mann-Whitney test	NTT vs. SE tissue	0.0017	0.176	0.45	0.0108	<0.0001	<0.0001	0.001	0.0171
	HV vs. preOP	0.6972	>0.9999	0.6922	0.6256	0.0027	0.0315	0.2366	0.7903
	HV vs. postOP	0.9973	0.3750	0.0674	0.0202	0.0038	0.0127	0.4333	0.6700
	RCLS vs. SE tissue	0.1129	-	0.001	-	0.0167	-	0.1467	-
Wilcoxon (matched-pairs signed rank test)	preOP vs. postOP	0.8386	>0.9999	0.1122	0.125	0.8497	0.6875	0.5042	>0.9999
	preOP vs. SE tissue	<0.0001	0.5000	<0.001	<0.0001	0.2363	<0.0001	0.0029	0.0016
Wilcoxon signed rank test	RCLS vs. SE tissue	-	0.0313	-	<0.0001	-	<0.0001	-	0.0020

NTT, non-malignant diagnoses; SE, seminoma.; HV, healthy volunteer.

© 2021 by the authors. Licensee MDPI, Basel, Switzerland. This article is an open access article distributed under the terms and conditions of the Creative Commons Attribution (CC BY) license (http://creativecommons.org/licenses/by/4.0/).
